# Evaluation on Preparation and Performance of a Low-Carbon Alkali-Activated Recycled Concrete under Different Cementitious Material Systems

**DOI:** 10.3390/ma17194869

**Published:** 2024-10-03

**Authors:** Cheng Liu, Xinyan Wang, Yujiao Li, Qiuyi Li, Gongbing Yue

**Affiliations:** College of Civil Engineering & Architecture, Qingdao Agricultural University, Qingdao 266109, China; 20222204027@stu.qau.edu.cn (C.L.); 20212204016@stu.qau.edu.cn (X.W.); liyujiao0212@163.com (Y.L.); lqyyxn@163.com (Q.L.)

**Keywords:** alkali-activated, mechanical properties, mix proportion design, recycled coarse aggregates, slag powder

## Abstract

A green, low-carbon concrete is a top way to recycle waste in construction. This study uses industrial solid waste slag powder (S95) and fly ash (FA) as binders to completely replace cement. This study used recycled coarse aggregate (RCA) instead of natural coarse aggregate (NCA). This is to prepare alkali-activated recycled concrete (AARC) with different cementitious material systems. Alkali-activated concrete (AAC) mixtures are modified for strength and performance based on the mechanical qualities and durability of AARC. Also, the time-varying effects of the environment on AARC properties are explored. The results show that with the performance enhancement of RCA, the mechanical performance of AARC is significantly improved. As RCA’s quality improves, so does AARC’s compressive strength. At a cementitious material content of 550 kg/m^3^, AARC’s 28d compressive strengths using I-, II-, and III-class RCA were reduced by 2.2%, 12.7%, and 21.8%, respectively. I-class AARC has characteristics similar to natural aggregate concrete (NAC) in terms of shrinkage, resistance to chloride penetration, carbonization, and frost resistance. AARC is a new type of green building material that uses industrial solid waste to prepare alkali-activated cementitious materials. It can effectively reduce the amount of cement and alleviate energy consumption. This is conducive to the reuse of resources, environmental protection, and sustainable development.

## 1. Introduction

Urbanization and economic growth have led to a lot of construction waste [1]. In China, it makes up about 40% of all solid waste [2]. For building rubbish, landfill and open-pile treatment have caused ecological damage. The development of new types of buildings has also led to the overuse of natural materials [3,4]. Thus, improving the use of solid waste and promoting the green treatment of construction waste are key issues in construction [5,6].

Construction waste is crumbled and sorted to prepare recycled aggregate. This is a widely accepted way to use construction waste. Recycled aggregates, unlike natural ones, can reduce the harm from raw material production. At the same time, this realizes the recycling of construction waste [7,8,9]. Wu et al. [10] found that a lower water/binder ratio (w/b) can improve the strength of recycled aggregate concrete (RAC). If the w/b ratio is below 0.45, the strength is about as strong as ordinary concrete. Liang et al. [11] found that using CO_2_-modified aggregate to make recycled concrete can improve its strength and durability. Li et al. [12] proved that a 10% FA content increases the recycled concrete’s compressive strength by 8.5%.

At present, recycled concrete uses ordinary cement as the cementitious system [13]. However, making ordinary cement consumes a lot of energy and emits CO_2_ [14]. This hinders green, low-carbon progress in the concrete industry [15]. Alkali-activated materials have high strength, a fast set, and great anti-corrosion properties. Alkali-activated cementitious materials come mainly from industrial solid waste. This waste has pozzolanic activity and some hydraulic properties [16,17]. Compared with traditional cement, alkali-activated cementitious materials have many advantages. These include rapid hardening, high strength, low heat, corrosion resistance, frost resistance, and high temperature resistance [18]. Alkali-activated cement can react with Ca(OH)_2_ [19]. It is a cement hydration product in recycled aggregate and can improve the quality of recycled materials [20]. Tang et al. [21] found that under the same curing conditions, alkali-activated FA recycled concrete at the same age had higher strength than ordinary recycled concrete. In this study, an S95 + FA composite system was used to compare with recycled concrete. Raghav et al. [22] found that activated FA promoted the growth of C-S-H, which was conducive to the formation of a solidified structure. The experimental study found that the incorporation of FA slowed down the rapid setting of alkali slag concrete to a certain extent. Vo et al. [23] showed that the incorporation of FA into alkali-activated slag concrete can prolong the setting time. In this way, the influence of a too-fast setting speed on the compactness of concrete is avoided. It is applied to the preparation of green new cementitious materials, which can effectively save resources and protect the environment and has broad application prospects. Therefore, the alkali-activated cementitious system is highly significant for cement reduction and industrial solid waste utilization in future applications [24,25].

This study used solid wastes, including S95 and FA, as cementitious materials. Water glass and NaOH were used as the alkali-activated solution. The AARC used different-class RCAs as its concrete aggregate. It was tested with different cementitious systems. Firstly, the effect of FA content on the mechanical property of alkali-activated mortar was discussed [26,27], and the optimum replacement ratio of FA suitable for preparing alkali-activated RAC was determined [28]. Then, the mechanical performance and endurance of AARC were systematically studied, and the mix proportion ratio of AARC with controllable performance was optimized. The preparation method of recycled concrete with a low-carbon cementitious material system was proposed to improve the greenness and high added value application of recycled aggregate and solid wastes [29]. In this paper, the performance differences between AARC and NAC are compared and analyzed. The effects of the types, composition, activator concentration, and recycled aggregate quality of alkali-activated cementitious materials on the microstructure characteristics, hydration products, and mechanical properties of AAC are systematically studied. Combined with the time-varying law of AARC durability in alkaline environment, the AARC mix ratio can be designed, and the AARC preparation method with controllable performance is proposed.

## 2. Experiment Program

### 2.1. Raw Material

**Figure 1:**
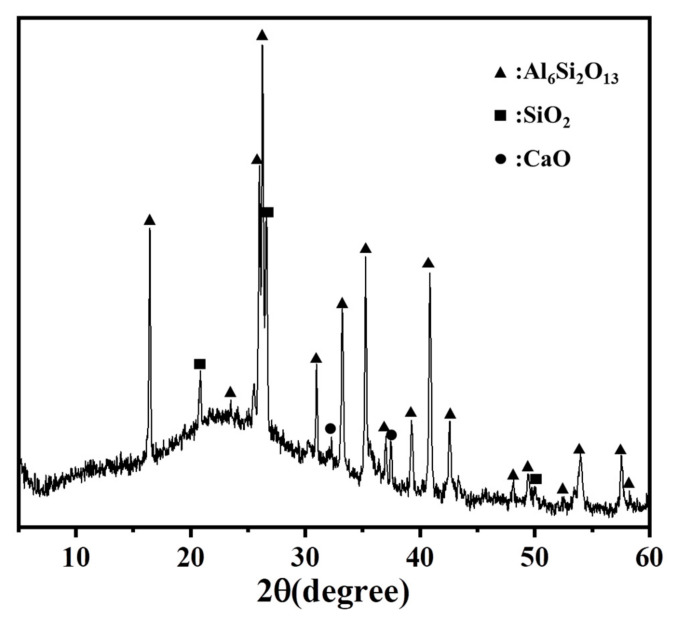
XRD analysis of FA.

In the present study, the S95-grade blast furnace S95 (Shanshui Cement Co., Ltd., Qingdao, China) was used. The activation coefficient was 0.41, and the alkaline modulus was 0.94. Unclassified low-calcium FA was used (Qingdao Qingjian New Materials Group Co., Ltd., Qingdao, China); its mineral composition is displayed in Table 1. Figure 1 shows the XRD analysis. In AAC, a mixture of NaSiO_3_ constitutes a basic initiator with a proportion of 1.53. The base/modulus ratio (Ms) is equivalent to 3.4 M (Ms represents the ratio of SiO_2_ to Na_2_O; Na_2_O = 3.4%, and SiO_2_ = 28.76% by mass) and commercially produced NaOH (3.0 M and 3.5 M) solution.

Natural fluvial sand served as fine aggregate, with a fineness modulus of 2.6 and a soil content of 0.7%. RCA (I-, II-, and III-class) and NCA were used as coarse aggregate for comparative analysis, respectively, and the performance indicators are displayed in Table 2.

### 2.2. Mix Proportion of Concrete

S95 + FA was used to replace cement as cementitious material. The replacement rates of FA were 0%, 15%, 30%, 45%, and 60%, respectively. Water glass with a ratio of 1.0 and alkali solution concentrations of 3.0% and 3.5% were selected. Water glass was preferred as a stimulant. The fine aggregate was made of natural river sand. The alkali activation mortar is shown in Table 3.

**Figure 2:**
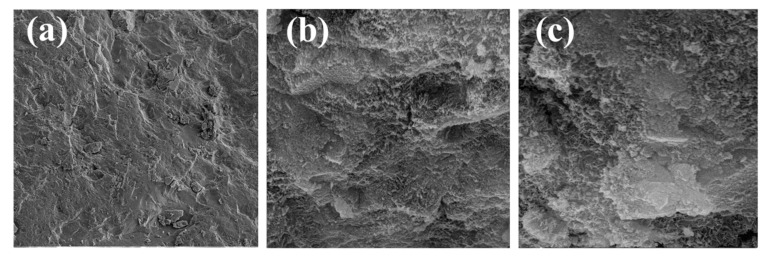
28 d SEM micromorphology of AAC (SF_1_-1): (**a**) 3000×; (**b**) 5000×; (**c**) 15,000×.

**Figure 3:**
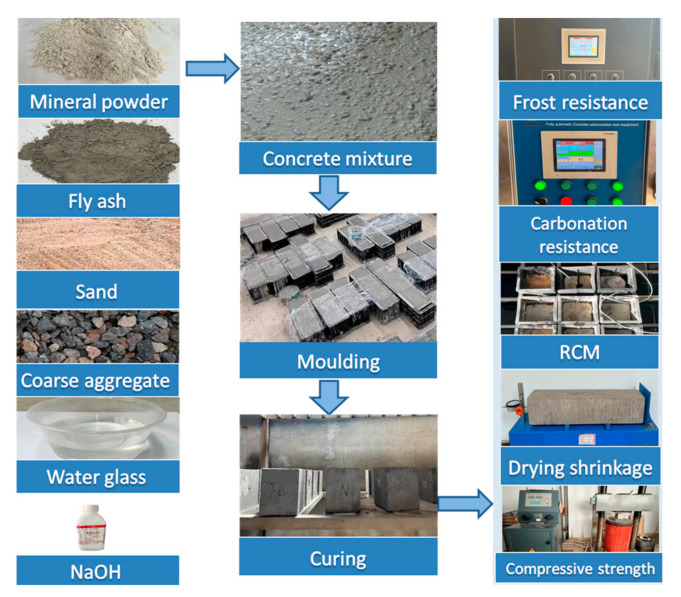
Test flowchart of AARC.

NCA and RCA were used as raw materials to prepare AAC and AARC. During the process of preparation, the water absorption effect of coarse aggregate in the concrete proportion should be considered to ensure the processability of the mixture [30]. Under the condition that the alkali concentration is content, the additional water increases the sol ratio by 0.02. There are many types of RCA quality, and its water absorption rate is inconsistent. The larger the content and the smaller the maximum particle size, the smaller the slump constant and compressive strength of the concrete [31]. In the process of preparing concrete, the actual amount of alkali solution is adjusted by slump. The mixture ratio design of AARC is shown in Table 4. SEM is used to inspect the microstructure of alkali-activated recycled cement paste at 3, 7, and 28 d. SEM images of the AAC are displayed in Figure 2. It can be seen from the diagram that the gel products are rich. It can be seen from Figure 2c that there is a dense three-dimensional network structure. The test flowchart is displayed in Figure 3.

### 2.3. Test Methods

According to the code “Standard for test methods of concrete physical and mechanical properties” (GB/T 50081-2019, [32]), three cubic specimens with the dimension of 150 mm × 150 mm × 150 mm were used for the compression strength test of recycled concrete at each curing age [33]. After demolding, the concrete specimen was moved to a standard curing room. The curing temperature was (20 ± 2) °C, and the relative humidity was in the range of (90 ± 5)%. Compressive strength tests for recycled concrete were carried out at the ages of 3, 7, 14, and 28 d.

From the perspective of durability, by means of the national standard “Test Methods for Long-term Performance and Durability Performance of Ordinary Concrete” (GB/T 50082-2009, [34]), the test for resistance to drying shrinkage of AARC was carried out. The research results meet the above standard requirements. The drying shrinkage rate of different recycled concrete at 1, 3, 7, 14, 28, 45, and 60 d was measured, respectively. The accuracy of drying shrinkage was determined to be 0.1%. Meanwhile, the accurate determination of resistance to chloride ion penetration of concrete is an important prerequisite for the durability design of reinforced concrete construction [35]. The chloride ion permeability test was carried out for the calculation of the chloride ion permeability coefficient. Moreover, the carbonization depth test for recycled concrete was conducted at 3, 7, 14, and 28 d [36]. The temperature of the carbonization chamber was set to (20 ± 3) °C. The humidity was in the range of (70 ± 3)%. The carbon dioxide concentration was controlled to (20 ± 2)%. Taking comprehensively the test conditions, cycles, and other factors into account, the freezing-and-thawing-cycle test of recycled concrete cured for 28 days was proceeded through the rapid-freezing method. Quality loss of the specimen was measured every 25 freezing-and-thawing cycles.

## 3. Results and Discussion

### 3.1. Optimum Content Determination of FA

**Figure 4:**
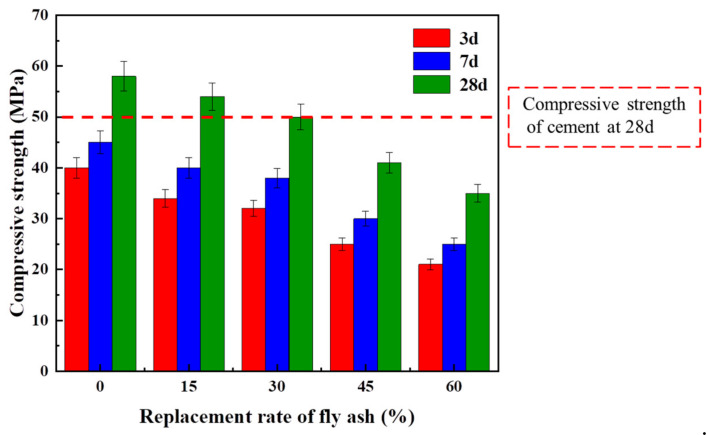
Compressive strength of mortar at different replacement rates of FA.

Figure 4 shows the effect of substitution rate of FA on the compressive strength of mortar. It can be seen that with the increasing substitution rate of FA, the compressive strength of mortar shows a downward trend at different curing ages. However, it does not destroy the characteristics of alkali-activated materials, with fast strength growth in the early stage and torpid growth in the later period. Bai et al. found that the reaction process of alkali-activated materials was fast in alkali-activated slag concrete, and the setting time of alkali-activated slag was shortened [37]. On this basis, S95 was used as cementitious material in the experiment. The setting time was alleviated by adding different amounts of FA.

Unlike the hydration process of traditional cement, more Ca(OH)_2_ products can be produced under the alkali-activated cementitious system, and it can react with SiO_2_ and Al_2_O_3_ in FA to generate calcium silicate hydrated (C-S-H) gel, calcium aluminosilicate hydrated (C-A-S-H) gel, and other products. This results in lower strength due to the incorporation of FA. In the early stage of hydration, mainly S95 participates in the hydration reaction to generate C-S-H gel, and a very small amount of FA participates in the reaction. With the continuous hydration reaction, the strength of FA mortar gradually increased in the later stage.

When the replacement rate is less than 30%, for every 15% increase in FA, the 28 d strength loss rate is 3%~4%. At a substitution rate of 30%, the 28-day intensity is lost by 7% compared to the control group. For all that, when the replacement rate is 45%, the intensity decreases by 10% compared to the replacement rate of 30%. When the replacement ratio of FA exceeds 30%, the intensity is significantly decreased sharply. In addition, under the S95 system, the setting speed of mortar was faster. The incorporation of FA can delay its setting time. Accordingly, in this experiment, it is found that the optimum replacement ratio of FA in the alkali-activated S95 + FA composite system is 30%, which can ensure workability and mechanical property requirements while keeping costs as low as possible.

### 3.2. Workability of Alkali-Activated Recycled Concrete

**Figure 5:**
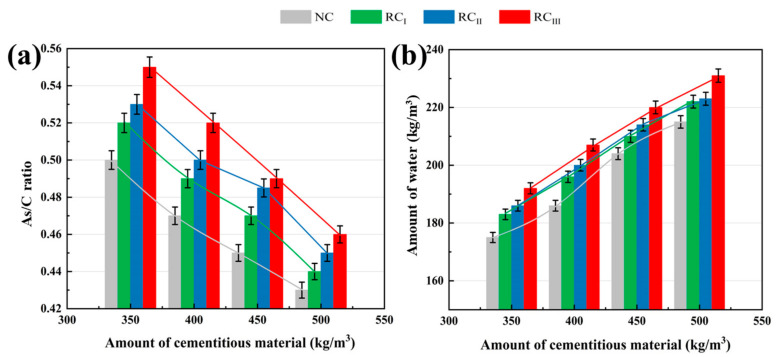
Relationship between the amount of alkali solution and cementitious material with different coarse aggregate. (NC—natural aggregate; RC_I_—I recycled coarse aggregates; RC_II_—II recycled coarse aggregates; RC_III_—III recycled coarse aggregates.)

Figure 5 presents the relationship between the amount of alkali solution and the amount of cementitious material with various coarse aggregates. It can be found that the amount of alkali-activated natural aggregate and recycled aggregate is proportional to the amount of cementitious material. Under the same amount of cementitious material, the water consumption and alkali solution of RCA concrete are significantly greater than that of NAC. This ensures that the concrete has the same workability [38]. With the quality improvement of RCA, the water consumption of the prepared concrete is reduced. Hameed et al. showed that cement substitution increased the water absorption of concrete samples [39]. However, RCA itself has a larger water absorption, and its water absorption is higher than that of NCA. In the process of concrete preparation, more water is needed to ensure that the mixture has good workability. Therefore, the high water absorption of RAC is to adjust the performance of RAC by adding additional water. The mixture ratio design is adjusted by using additional water diversion. In comparison to the natural aggregates, the surface of RCA has old hardened mortar and cracks generated inside [40], causing the water absorption performance of RCA to increase to a certain extent. I-class and II-class recycled aggregates that have undergone primary and secondary physical strengthening, respectively, have relatively fewer old mortars attached to the outside than III-class recycled aggregates. The fundamental characteristic of RCA is significantly improved, which effectively reduces the water consumption of the concrete mixture. In order to ensure that the concrete has the same workability, the water consumption of concrete mixtures of different aggregate types is: III-class recycled aggregate > II-class recycled aggregate > I-class recycled aggregate > natural aggregate.

### 3.3. Mechanical Property of AARC

As the cementitious material content increases due to the increment of hydration products, the inner architecture of concrete is more densified, which is beneficial for strength development. The 28 d compressive strength of different recycled concrete gradually increases, which has a good linear correlation. Meanwhile, the alkaline components in the alkali-activated cementitious material system can react with Ca(OH)_2_ and other products in the residual old mortar on the outer surface of recycled aggregate. Ca^2+^ provided by Ca(OH)_2_ products hydrolysis accelerates the formation of C-S-H gel, which has an effect on improving the performance of recycled aggregate. The increase of cementitious material has the most remarkable influence on the strength of III-class RAC. The cementitious system is the key factor affecting strength development.

**Figure 6:**
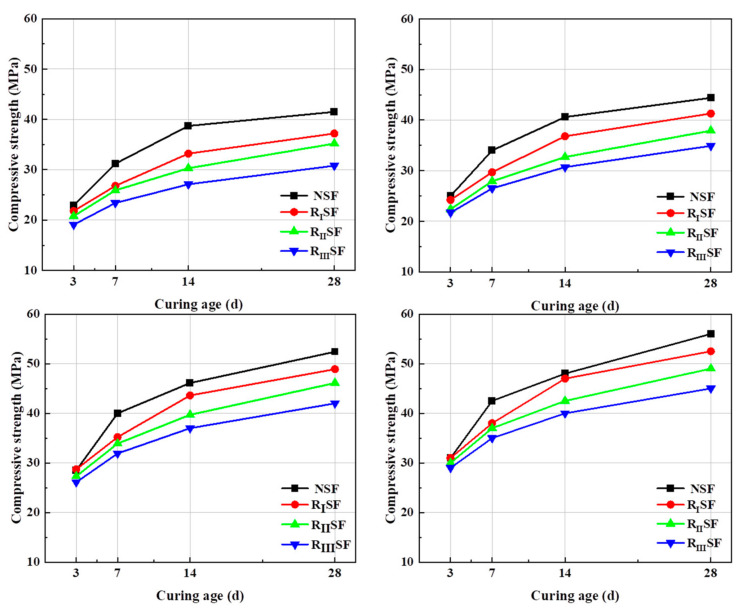
The compressive strength of concrete at cementitious materials.

Figure 6 shows the variation of compressive strength of recycled concrete with curing age under the condition of different gel material content. The 28 d compressive strength of NAC under the S95 + FA composite system reaches 40 MPa at 350 kg/m^3^ of cementitious material. In contrast, the 28 d compressive strength of I-class RAC is 37 MPa, and the 28 d compressive strength of III-class RAC is only 29 MPa. However, when the amount of cementitious material is 550 kg/m^3^, the 28 d compressive strength of NAC can reach 51 MPa. Under the same conditions, the 28 d compressive strength of I-class RAC is 46 MPa, and the 28 d compressive strength of III-class RAC is 38 MPa. With the quality improvement of RCA, the compressive strength of AARC is also correspondingly improved. The compressive strength of AARC prepared from III-class RCA is much smaller than that of AAC. This is because during the preparation process, there are many micro-cracks inside the RCA. A certain amount of old mortar is attached to the aggregate surface [41], which makes the water absorption ratio of recycled aggregate higher than that of natural aggregate. The water glass, as liquid phase in AAC, can enter the inside of the adhering old mortar through micro-cracks in the aggregate, causing the performance of recycled aggregate to improve to a certain extent. Meanwhile, Ca(OH)_2_ existing in recycled aggregate easily reacts with alkaline components in water glass. The produced CaCO_3_ covers the outside of recycled aggregate, which improves the quality of recycled aggregate. According to Moussadik et al. [42], high compressive strength is obtained by alkali solution. In the experiment, the alkali concentration was used for verification. The specific amount of alkali solution was adjusted by slump. The alkali solution excites S95, and the performance of the cementitious material is enhanced. The strength of the test block is improved. However, the partial penetration of water glass will reduce the activation degree of S95 and FA.

### 3.4. Mix Proportion Ratio Optimization

#### 3.4.1. Confirmation of Base Mix Proportion Ratio

The research methods and requirements were strictly in accordance with the relevant national standards of China’s ‘Technical Standard for the Application of Alkali Slag Concrete’ (JGJ/T 439-2018, [43]), ‘Design Regulations for the Mix Ratio of Normal Concrete’ (JGJ 55-2011, [44]), and ‘Technical Regulations for the Application of Recycled Aggregates’ (JGJ/T 240-2011, [45]). The standard deviations of ordinary concrete and RAC were chosen to represent the standard deviations of AAC and AARC (as shown in Table 5), respectively. Since the design strength grades of the concrete are all less than C60, the preparation strength is determined by the following expression:

f_(cu,0) ≥ f_(cu,k) + 1.645^*σ*^ (1)

where f_(cu,0) is the preparation strength (MPa); f_(cu,k) is the standard value for the compressive strength of the concrete cube (the value of the concrete design strength grade is taken in this test) (MPa); and σ is the standard variance of concrete strength (MPa).

The 28 d compressive strength of different AACs is used as the basis for concrete strength design. Combined with the linear fitting relationship in Table 6 and Table 7, the amount of cementitious material and alkali solution of recycled concrete with different strength grades under the two cementitious systems are calculated. According to the amount of cementitious material and 3.5% alkali concentration, the dosage of water glass and sodium hydroxide is calculated, and the mix proportion ratio is finally determined. Compared with traditional cement-based concrete, the mix proportion ratio of AARC prepared from I-class RCA is the best. The reference mixture proportion ratio of AAC produced from natural aggregate and I-class recycled aggregate can obtain the optimized S95 and FA composite cementitious material system. The reference mix proportion ratio of AAC with different coarse aggregates is systematically studied, as shown in Table 8.

#### 3.4.2. Verification of Reference Ratio Strength

On the basis of the improved mixture ratio in Table 8, different AACs were prepared. The cube test specimens with the size of 100 mm × 100 mm × 100 mm were used for compressive strength. The concrete prepared on the basis of reference mix ratio meets the design requirements.

**Figure 7:**
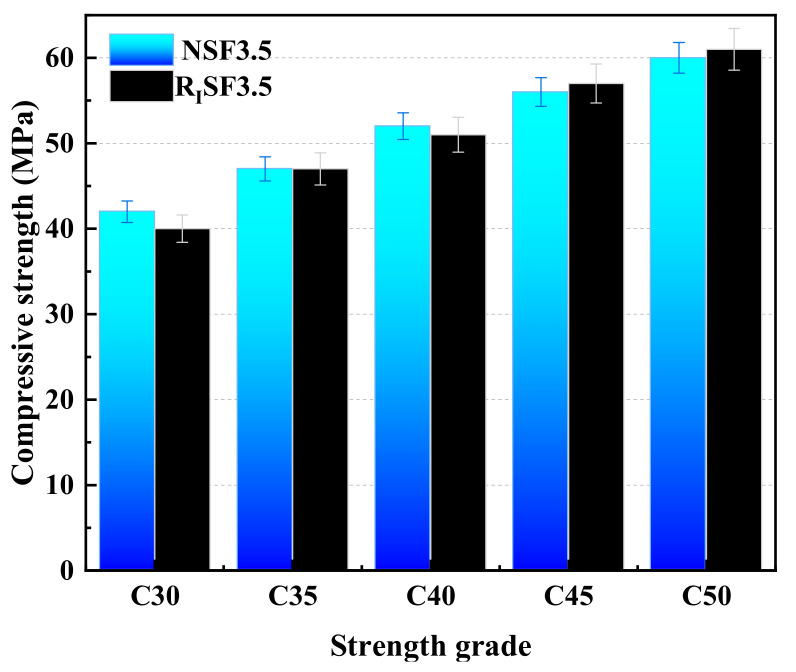
28 d measured strength values for concrete of different strength grades.

Figure 7 presents the measured 28 d strength values of different strength-grade concrete prepared according to the reference mix proportion ratio. It can be observed that under the two cementitious material systems, the AAC prepared from natural aggregate and I-class recycled aggregate meets the design specifications of each strength grade. Therefore, the reference mixture proportion ratio of AAC on strength design has high practicability.

### 3.5. Durability of Recycled Concrete

#### 3.5.1. Shrinkage of Different Concrete

**Figure 8:**
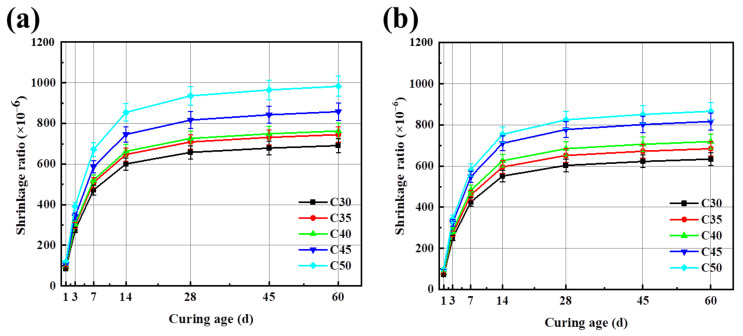
(**a**) Shrinkage of AAC concrete with curing age; (**b**) Shrinkage of AARC concrete with curing age.

Figure 8 shows the shrinkage ratios of different strength-grade concrete with curing age. The results reveal that the incorporation of FA improved the workability of the concrete mix [46]. It also effectively improves the shrinkage performance of AAC. The higher the strength level of concrete and the more cementitious material used, the greater the shrinkage rate. Due to the relatively low activity and the smooth surface of FA, the incorporation of FA plays a complementary role in the particle gradation of cementitious materials, which can effectively fill the voids inside the concrete. The incorporation of FA also displaces some of the moisture in the void, which can further alleviate the hydration reaction and greatly inhibit the shrinkage of alkali-activated cementitious material.

#### 3.5.2. Chloride Ion Permeability of Different Types of Concrete

**Figure 9:**
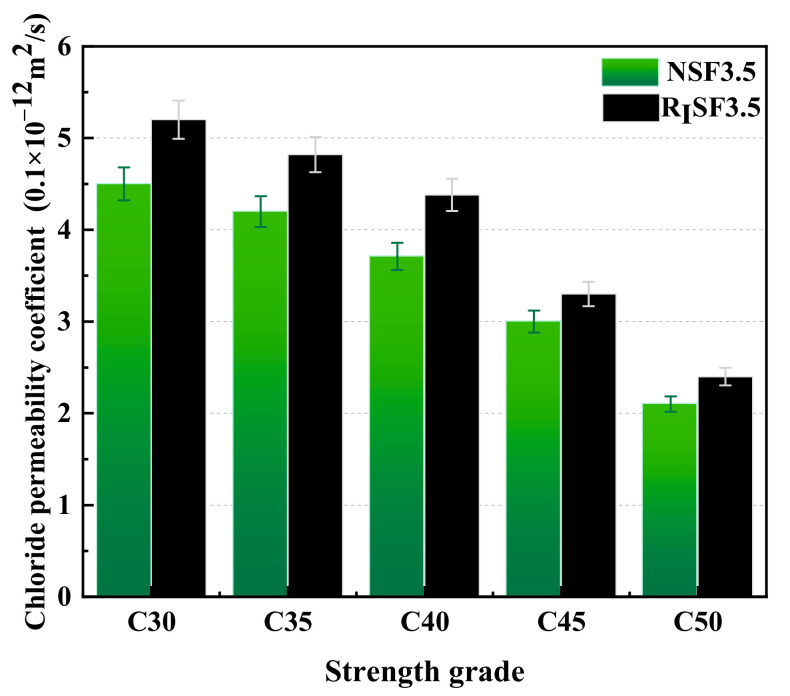
Relationship between chloride penetration coefficient and strength grade at 28 d.

From Figure 9, the relation between the chloride ion penetration coefficient and the strength grade for various types of concrete can be seen. It can be learned that the chloride ion penetration coefficients of AAC and AARC both decrease with the enhancement of strength grade. The higher the strength grade of concrete, the more cementitious materials it contains, the smaller the internal porosity, and the higher the compactness [47]. The chloride resistance penetrating quality of strong concrete is much better than that of low-strength concrete. Meanwhile, the resistance to chloride ion permeability of AAC and AARC under the S95 system is obviously better than that of the S95–FA composite system. Bayraktar et al. found that the use of FA made concrete more permeable to chloride ions [48]. In-depth scientific research on the durability of RAC was carried out. It was found that FA can improve resistance to chloride ion erosion. The increase in the content of cementitious materials makes the internal structure of concrete more dense. The passage of chloride ions into the concrete is blocked. The hydration products of alkali-activated S95 are mainly C-(A)-S-H. The hydration products of alkali-activated FA are mainly N-A-S-H. There are a large amount of gel pores inside, causing that the overall structure to be relatively loose. The FA content in the S95–FA composite system is 30%; the system produces C-N-A-S-H gels. Therefore, the S95 system is better at resisting chloride ion penetration than the composite system.

For different aggregate types, the chloride permeability coefficient of AAC is less than that of AARC under the same cementitious system. The properties of I-class recycled aggregates are relatively similar to those of natural aggregates. Therefore, the influence of aggregate type on the chloride ion penetration resistance performance of concrete is less than the influence of cementitious systems [49]. In the powder–FA composite system, the chloride permeability coefficient of AAC is 12.2% smaller than that of AARC. There are various complex interface structures in recycled concrete. The interface structure is loose and rich in Ca(OH)_2_. It provides access to aggressive media such as chlorine salts, which can lead to the deterioration of concrete.

#### 3.5.3. Carbonization Resistance of Different Concrete

**Figure 10:**
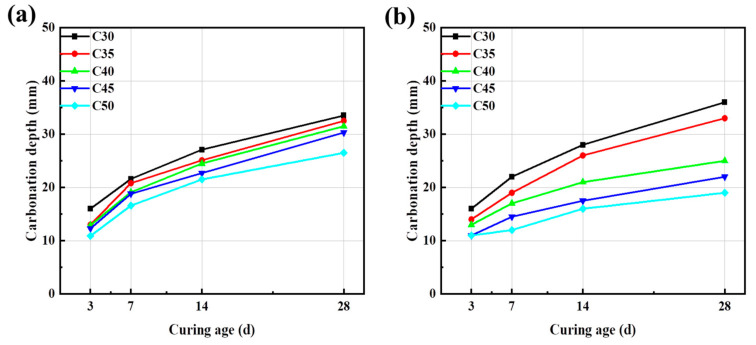
(**a**) Carbonization depth of AAC concrete of S95 + FA system with age; (**b**) carbonization depth of AARC concrete of S95 + FA system with age.

The carbonization specimens of AARC are made of 100 mm × 100 mm × 400 mm prisms, and the carbonization depth is tested at 3, 7, 14, and 28 d, respectively. Under conditions of different strength grades, the correlation between the carbonization depth and the curing age of different concrete is shown in Figure 10. During the carbonization cycle, the growth rate of 7 d carbonization depth is significantly faster than that of later carbonization. The carbonization trend is faster and then slow [50]. Under the two cementitious materials, the carbonization resistance of AAC and AARC increases with the increase of strength grade. This is mainly because the higher the concrete’s strength grade, the more cementitious material, and the more hydration products are formed, making the concrete’s internal structure denser. Meanwhile, the carbonization depth of AARC is higher than that of AAC at each age of a certain strength grade. Carbonization depths of AAC and AARC at 28 d are up to 33.5 mm and 36.0 mm, respectively, which is mainly due to the existing microcracks and poor grading inside recycled aggregate in concrete.

**Figure 11:**
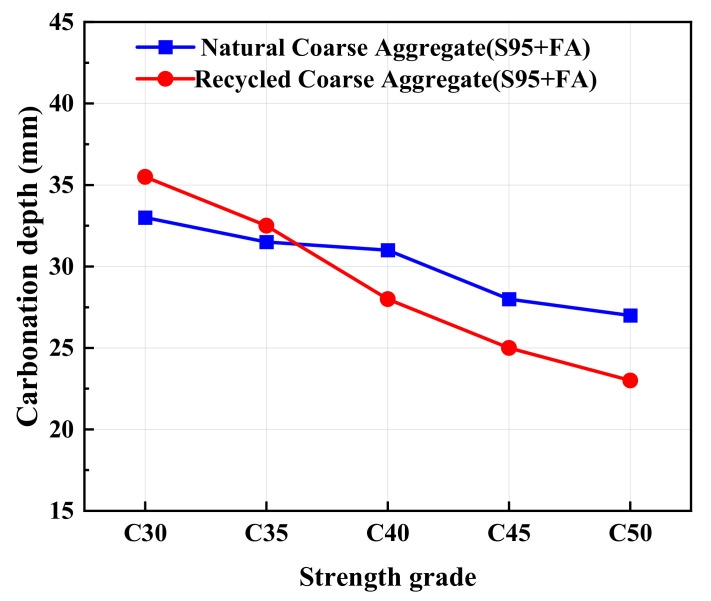
28 d carbonization depth value of composite system.

Figure 11 presents the effect of different aggregate types on carbonization depth. It can be seen that as the concrete strength grade is lower than C35, the carbonization depth of AARC is higher than that of AAC. On the contrary, when the grade exceeds C35, the carbonization depth of AARC is less than that of AAC. The increment of cementitious materials can more fully wrap the recycled aggregate and reduce the performance defects of the recycled aggregate. Meanwhile, the prolongation of curing time makes the carbonization resistance of AARC better than that of AAC. Conversely, low-strength concrete cannot resist the weakening effect of its own inferiority. Therefore, AARC in low-strength-grade concrete has lower carbonization resistance than AAC. Martinez et al. used waste to enhance carbonation resistance to improve performance and compressive strength [51]. The carbonation resistance of AARC is worse than that of ordinary cement concrete. The addition of FA effectively improves the performance of AARC. The carbonation resistance of hydration products in the composite system is beneficial to resist the entry of CO_2_ and water. The carbonation resistance of AAC and AARC is of great significance for the application and promotion of this material in practical engineering.

#### 3.5.4. Frost Resistance of Different Concrete

**Figure 12:**
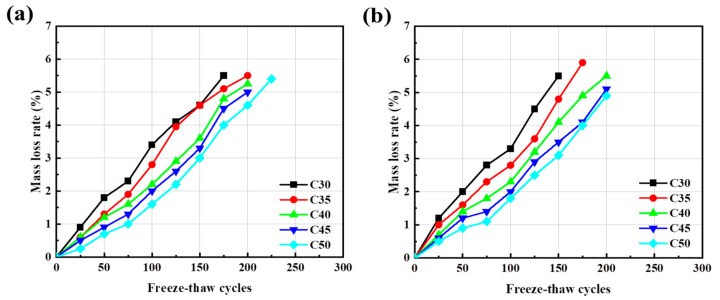
(**a**) Relationship between the mass loss rate of AAC and freeze–thaw cycles; (**b**) relationship between the mass loss rate of AARC and freeze–thaw cycles.

Figure 12 shows the correlation between rate of quality-led loss and freezing-and-thawing cycles. Taking the S95 + FA system AARC as an example, it can be seen that when the freezing-and-thawing cycle adds up to 100 times, the quality loss rates of C30, C35, C40, C45, and C50 concrete of each strength grade are 3.22%, 2.75%, 2.25%, 2.05%, and 1.79%, respectively. As the strength grade of concrete increases, its frost resistance improves. For the same number of freeze–thaw cycles, the mass loss rate of low-strength concrete is higher than that of high-strength concrete.

**Figure 13:**
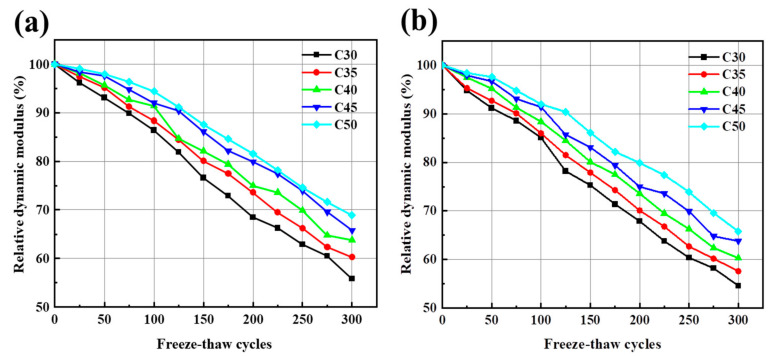
(**a**) Relationship between the relative dynamic modulus of elasticity of AAC and freeze–thaw cycles; (**b**) relationship between the relative dynamic modulus of elasticity of AARC and freeze–thaw cycles.

From Figure 13, with an increase of the number of freeze–thaw cycles, the relative dynamic elastic modulus of various concretes decreases. At the initial stage of the freezing-and-thawing-cycle test, the relative kinetic modulus changes by a small margin. As the freezing-and-thawing cycle increases, the loss rate of the relative kinetic modulus of elasticity also increases. Zheng et al. [52] showed that under the action of freeze–thaw cycles, the number of freeze–thaw cycles was positively correlated with mass loss and negatively correlated with relative dynamic elastic modulus. Due to the old hardened paste and microcracks, the water absorption ratio of RCA is greater than that of NCA, and the recycled aggregate contains a certain amount of freestream flow water inside. Under a low-temperature environment, the water inside the aggregate freezes and causes volume expansion, destroying the interface among the aggregate and the slurry [53], which makes AARC more susceptible to destruction than AAC under the same freeze–thaw conditions.

## 4. Conclusions

In this study, under different cementitious material systems, the multiple industrial solid waste and RCA were used to prepare a low-carbon AARC. The obtained key conclusions are as follows:

(1) Through the analysis of the mechanical properties and microstructure of alkali-activated mortar, the concentration and modulus of activator were adjusted to make the 28 d compressive strength consistent with the strength of cement mortar. The optimum content of FA was determined to be 30%, and the solid waste-based alkali-activated cementitious material system was optimized.
(2) Based on the mechanical properties of AARC with different aggregate quality, the linear relationship between 28 d compressive strength and alkali-activated cementitious material system and sol ratio was established, and the mix ratio of AARC with different strength grades was optimized.
(3) The 28 d compressive strength of AARC after optimization can meet the standard requirements. Its shrinkage performance, chloride ion penetration resistance, and frost resistance are similar to NAC, but the carbonation resistance of AARC above strength grade C40 is significantly improved.

The alkali-activated cementitious system was optimized, and the mix ratio of AARC with different strength grades was determined. The durability of AARC with different strength grades was systematically studied, and the preparation technology with controllable performance was proposed. The ultimate goal of the research is to save cement and alleviate the serious shortage of natural resources in order to maintain the sustainable development of the construction industry and the application of green recycled concrete.

## Figures and Tables

**Table 1:** XRF analysis for mineral composition of FA (%).

SiO_2_	Al_2_O_3_	Fe_2_O_3_	CaO	Na_2_O	MnO	MgO	SO_3_	TiO_2_	BaO	SrO
56.12	30.25	5.65	4.59	0.38	0.05	0.43	0.91	1.43	0.07	0.12

**Table 2:** Performance indicators of coarse aggregate.

Projects	Standard Regulations				Recycled Coarse Aggregates		
	NC	I	II	III	I	II	III
Micro-powder content (%)	<0.5	<1.0	<2.0	<3.0	0.6	1.4	1.8
Water absorption rate (%)	<2	<3.0	<5.0	<8.0	2.3	4.2	7.0
Needle flake particle content (%)	<8	<10.0			1.7	3.4	6.2
Impurity content (%)	<1	<1.0			0.2	0.7	0.8
Robustness (%)	<5	<5.0	<10.0	<15.0	3.8	7.4	14.5
Crushing index (%)	<10	<12.0	<20.0	<30.0	9.5	18.0	28.7
Apparent density (kg/m^3^)	>2500	>2450	>2350	>2250	2469	2452	2313
Void ratio (%)	<45	<47.0	<50.0	<53.0	42.0	44.0	45.0

Note: NC—natural aggregate; class I—I recycled aggregates; class II—II recycled aggregates; class III—III recycled aggregates.

**Table 3:** Mix proportion of AARC.

Mark	Replacement Ratio (%)	Alkali Equivalent (%)	Modulus	Mineral Powder (kg/m^3^)	FA (kg/m^3^)	Sand (kg/m^3^)	As/C Ratio
FA-0	0	3.5	1.00	400	0	724	0.45
FA-15	15	3.5	1.00	340	60	724	0.45
FA-30	30	3.5	1.00	280	120	724	0.45
FA-45	45	3.5	1.00	220	180	724	0.45
FA-60	60	3.5	1.00	160	240	724	0.45

Note: As/C ratio: Alkali solution/cementitious materials ratio.

**Table 4:** The mix proportion design of AARC (kg/m^3^).

Mark	Mineral Powder	Fly Ash	Alkali Equivalent	Sand	Coarse Aggregate	Water Glass	NaOH
SF_1_-1	245	105	3.0	742	1113	36.46	9.57
SF_1_-2	280	120	3.0	724	1086	41.67	10.93
SF_1_-3	315	135	3.0	706	1059	46.88	12.30
SF_1_-4	350	150	3.0	688	1032	52.09	13.66
SF_2_-1	245	105	3.5	742	1113	42.54	11.16
SF_2_-2	280	120	3.5	724	1086	48.62	12.75
SF_2_-3	315	135	3.5	706	1059	54.69	14.35
SF_2_-4	350	150	3.5	688	1032	60.77	15.94

Note: The coarse aggregate includes NCA, I-class RCA, II-class RCA, and III-class RCA; SF: S95 + FA.

**Table 5:** Standard deviation recommended values of compressive strength of AANA and RAC.

Strength Grade	≤C20	C25–C45	C50–C55
Standard value (σ)	4.0	5.0	6.0

**Table 6:** Linear fitting relationship between compressive strength and cementitious material content under different aggregate types.

Aggregate Type	Cementitious System	Regression Equation	Correlation Coefficient
Natural aggregate	S95 + FA	y = 0.1084x + 3.03	R^2^ = 0.9817
I-class RCA	S95 + FA	y = 0.1148x − 3.49	R^2^ = 0.9813
II-class RCA	S95 + FA	y = 0.1080x − 3.15	R^2^ = 0.9792
III-class RCA	S95 + FA	y = 0.1102x − 8.21	R^2^ = 0.9851

**Table 7:** Linear fitting relationship between compressive strength and sol ratio of concrete under different aggregate types.

Aggregate Type	Cementitious System	Regression Equation	Correlation Coefficient
Natural aggregate	S95 + FA	y = −227.9439x + 154.80	R^2^ = 0.9760
I-class RCA	S95 + FA	y = −217.6471x + 149.77	R^2^ = 0.9482
II-class RCA	S95 + FA	y = −200.8824x + 140.51	R^2^ = 0.9442
III-class RCA	S95 + FA	y = −183.6667x + 131.38	R^2^ = 0.9851

**Table 8:** Mix proportion ratio of AARC (kg/m^3^).

Mark	Strength Grade	S95	FA	Fine Aggregate	Coarse Aggregate	Water Glass	NaOH	Additional Water	Solubility Ratio
NSF	C30	227.1	97.3	743.8	1115.8	39.4	10.3	116.1	0.51
	C35	259.4	111.2	723.2	1084.8	45.0	11.8	124.5	0.49
	C40	291.7	125.0	703.4	1055.1	50.6	13.3	130.9	0.47
	C45	324.0	138.8	684.4	1026.5	56.3	14.8	135.2	0.45
	C50	367.2	157.4	658.8	988.2	63.8	16.7	137.8	0.42
R_I_SF	C30	254.2	108.9	716.3	1074.4	44.1	11.6	130.4	0.51
	C35	284.7	122.0	697.7	1046.5	49.4	13.0	136.7	0.49
	C40	315.2	135.1	679.8	1019.8	54.7	14.4	141.0	0.47
	C45	345.7	148.1	662.8	994.2	60.0	15.7	143.3	0.44
	C50	386.5	165.7	639.9	959.9	67.1	17.6	143.2	0.41

## Data Availability

Since the experiment was completed with the support of Qingdao Agricultural University, the data used to support the results of this study are available from the responsible person and the author upon request.

## References

[1] Feng C.H., Huang Y.H., Cui B.W., Zhu J., Li D., Guo H. (2022). Research progress on strengthening methods of building recycled aggregates. Mater. Rep..

[2] Yang H., Xia J. (2017). Urban construction and demolition waste and landfill failure in Shenzhen, China. Waste Manag..

[3] Wang C.Q., Yu L., Zhang J.J. (2024). Production forecast, comprehensive utilization, management measures and visualization analysis of construction waste. Sustain. Chem. Pharm..

[4] Sun Z., Gao Y., Yang J.J., Chen Y.X., Guo B.H.W. (2024). Development of urban building energy models for Wellington city in New Zealand with detailed survey data on envelope thermal characteristics. Energy Build..

[5] Guo H., Shi C.J., Guan X.M., Zhu J.P., Ding Y.H., Zhang H.B. (2018). Durability of recycled aggregate concrete: A review. Cem. Concr. Compos..

[6] Kabirifar K., Mojtahedi M., Wang C.X., Tam V.W.Y. (2020). Construction and demolition waste management contributing factors coupled with reduce, reuse, and recycle strategies for effective waste management: A review. J. Clean. Prod..

[7] Pan Y., Wang L.L., Chen B., Zhang J., Cheng G., Zhao Y. (2023). Study on the influence of impurities in recycled aggregate of construction waste on its performance in preparing recycled concrete. Sichuan Environ..

[8] Gu L.G., Liu Y., Zeng J.J., Zhang Z.Y., Pham P.N., Liu C., Zhuge Y. (2024). The synergistic effects of fibres on mechanical properties of recycled aggregate concrete: A comprehensive review. Constr. Build. Mater..

[9] Ding Z.K., Yang Q.F., Zhang Z.Y., Chen J.Y. (2024). Interfering implicit attitudes of adopting recycled products from construction wastes. J. Clean. Prod..

[10] Wu J.C. (2022). Application and promotion of recycled aggregate of construction waste in green concrete. Build. Technol. Dev..

[11] Liang C., Ma Z. (2020). Utilization of CO_2_ curing to enhance the properties of recycled aggregate and prepared concrete: A review. Cem. Concr. Compos..

[12] Wang X., Yan Y.R., Tong X.F., Gong Y.F. (2022). Investigation of Mineral Admixtures on Mechanical Properties of Alkali-Activated Recycled Concrete Powders Cement. Buildings.

[13] Liu X.L. (2022). Research progress of construction waste treatment and resource recycling. Leather Prod. Environ. Prot. Sci. Technol..

[14] Lippiatt N., Ling T.C., Pan S.Y. (2020). Towards carbon-neutral construction materials: Carbonation of cement-based materials and the future perspective. J. Build. Eng..

[15] Wang H.R. (2023). Study on heat flow model of fly ash and sodium hydroxide alkali activation: A case study of alkali activated cement. Real Estate World.

[16] Jin Z.Q., Zhao X., Du Y.J., Yang S.Y., Wang D.Q., Zhao T.J., Bai Y. (2022). Comprehensive properties of passive film formed in simulated pore solution of alkali-activated concrete. Constr. Build. Mater..

[17] Heikal M., Abd El Aleem S., Morsi W.M. (2016). Durability of composite cements containing granulated blast-furnace slag and silica nano-particles. Indian J. Eng. Mater. Sci..

[18] Zheng W.Z., Wu M.N., Wang Y. (2019). Research progress of alkali-inspired cementitious materials. J. Build. Struct..

[19] Yu S.Y., He J., Sang G.C., Yang S.Q., Liu G.Y. (2024). Study on hydration process of alkali-activated slag cement activated by weakly alkaline components. Constr. Build. Mater..

[20] Chen P., Wang Z.X., Cao S.J., Rong X., Shi Z.Y., Wang H. (2023). Study on axial compressive stress-strain relationship of alkali-activated slag lightweight aggregate concrete. Constr. Build. Mater..

[21] Tang L., Zhang H.G., Huang Q., Wang Q., Shi X.S. (2015). Study on sulfate resistance of fly ash base polymer recycled concrete. J. Sichuan Univ. (Eng. Sci. Ed.).

[22] Raghav M., Park T., Yang H.M., Lee S.Y., Karthick S., Lee H.S. (2021). Review of the Effects of Supplementary Cementitious Materials and Chemical Additives on the Physical, Mechanical and Durability Properties of Hydraulic Concrete. Materials.

[23] Vo D.H., Thi K.D.T., Hwang C.L., Liao M.C., Hsu W.L., Yehualaw M.D. (2023). Mechanical properties of concrete produced with alkali-activated slag-fly ash and recycled concrete aggregate and designed using the densified mixture design algorithm (DMDA) method: Effects of recycled aggregate content and alkaline solution. Dev. Built Environ..

[24] Aldandooh M.A.A., Bunnori N.M., Johari M.A.M., Jamrah A., Alnuaimi A. (2016). Retrofitting of damaged reinforced concrete beams with a new green cementitious composites material. Compos. Struct..

[25] Zhang X.Y., Yu R., Zhang J., Shui Z. (2022). A low-carbon alkali activated slag based ultra-high performance concrete (UHPC): Reaction kinetics and microstructure development. Journal of Cleaner Production.

[26] Song W.L. (2021). Analysis of the development direction and innovative ideas of construction management of construction engineering. Jiangxi Build. Mater..

[27] Wei Z.F., Wei Y.F. (2023). Application study on inhibition of alkali active aggregate. Sichuan Hydroelectr. Power.

[28] Hsu S., Chi M., Huang R. (2018). Effect of fineness and replacement ratio of ground fly ash on properties of blended cement mortar. Constr. Build. Mater..

[29] Li J., Deng X., Lu Z.Y., Li X.Y., Hou L., Jiang J., Yang F.Y., Zhang J.J., He K.W. (2024). Recycled concrete fines as a supplementary cementitious material: Mechanical performances, hydration, and microstructures in cementitious systems. Case Stud. Constr. Mater..

[30] Wang J.H., Huang Y., Yang G.T., Wei Q.A., Liu W.Z. (2022). Research progress on compressive properties of recycled concrete. Mater. Rep..

[31] Lee J.S., Kim E.S., Jang K.P., Park C.K., Kwon S.H. (2022). Prediction of concrete pumping based on correlation between slump and rheological properties. Adv. Concr. Constr..

[32] (2019). Standard for test methods of concrete physical and mechanical properties.

[33] Kou S.C., Zhan B.J., Poon C.S. (2012). Feasibility study of using recycled fresh concrete waste as coarse aggregates in concrete. Constr. Build. Mater..

[34] (2009). Standard for test methods of long-term performance and durability of ordinary concrete.

[35] Yu Z.X., Li Q.Y., Su D.L. (2022). Research progress on self-generated shrinkage and drying shrinkage properties of recycled concrete. Concrete.

[36] Yu Q., Wan X.M., Zhao T.J., Wang T., Han X., Sun Z.T. (2022). Study on chloride ion permeability resistance and electrical test method of alkali-excited slag concrete. Mater. Rep..

[37] Bai W.F., Ye D.Q., Ye S., Yuan C.Y., Guan J.F., Yang G., Xie C.P. (2024). Study on mechanical properties and damage mechanism of alkali-activated slag concrete. J. Build. Eng..

[38] Wang D.P., Chen P.Y., Wang L., Li X.K., Shen Q.H. (2018). Study on shrinkage characteristics of fly ash content on alkali-excited slag mortar. Bull. Chin. Ceram. Soc..

[39] Hameed R., Tahir M., Abbas S., Sheikh H.U., Kazmi S.M.S., Munir M.J. (2024). Mechanical and Durability Characterization of Hybrid Recycled Aggregate Concrete. Materials.

[40] Liu X., Wu J., Zhao X., Yan P.P., Ji W.Y. (2021). Effect of brick waste content on mechanical properties of mixed recycled concrete. Constr. Build. Mater..

[41] Wang Y.Y., Yuan H., Chen S.Y. (2022). Study on the ratio of alkali-inspired cementitious materials prepared from solid waste. Chem. Miner. Process..

[42] Moussadik A., El Fadili H., Saadi M., Diouri A. (2024). Lightweight aerated concrete based on activated powders of coal gangue and fly ash. Constr. Build. Mater..

[43] (2018). Technical standard for application of alkali-activated slag concrete.

[44] (2011). Specification for mix proportion design of ordinary concrete.

[45] (2011). Technical specification for application of recycled aggregate.

[46] Gan W. (2022). Study on the effect of fly ash content on the properties of recycled concrete of construction waste. West. Transp. Sci. Technol..

[47] Yue G.B., Ma Z.M., Liu M., Liang C.F., Ba G.Z. (2020). Damage behavior of the multiple ITZs in recycled aggregate concrete subjected to aggressive ion environment. Constr. Build. Mater..

[48] Bayraktar O.Y., Eshtewi S.S.T., Benli A., Kaplan G., Toklu K., Gunek F. (2021). The impact of RCA and fly ash on the mechanical and durability properties of polypropylene fibre-reinforced concrete exposed to freeze-thaw cycles and MgSO4 with ANN modeling. Constr. Build. Mater..

[49] Mi R.J., Pan G.H. (2020). Research progress on carbonization resistance of recycled concrete. J. Harbin Eng. Univ..

[50] Zhang S.F., Qi H.J. (2021). Research progress on durability of recycled concrete. Concrete.

[51] Martinez-Molina W., Chavez-Garcia H.L., Perez-Lopez T., Alonso-Guzman E.M., Arreola-Sanchez M., Navarrete-Seras M.A., Borrego-Perez J.A., Sanchez-Calvillo A., Guzman-Torres J.A., Perez-Quiroz J.T. (2021). Effect of the Addition of Agribusiness and Industrial Wastes as a Partial Substitution of Portland Cement for the Carbonation of Mortars. Materials.

[52] Zheng X.C., Liu F., Luo T., Duan Y.F., Yi Y., Hua C. (2021). Study on Durability and Pore Characteristics of Concrete under Salt Freezing Environment. Materials.

[53] Wu J.C., Cheng Y.D., Zhong T., Lai D.J., Chen Z.W. (2022). Review of research on the strengthening of recycled aggregates for construction waste. Environ. Sanit. Eng..

